# Modified gastric peroral endoscopic myotomy for helical stenosis after sleeve gastrectomy

**DOI:** 10.1016/j.vgie.2026.02.011

**Published:** 2026-03-03

**Authors:** Michael Lajin, Helen Sohn, Jon Ellison, Scott Honowitz

**Affiliations:** Sharp HealthCare, San Diego, California, USA

## Abstract

**Background and Aims:**

Gastric sleeve stenosis (GSS) occurs in 0.7% to 4% of cases after laparoscopic sleeve gastrectomy and can lead to leaks, fistulas, refractory reflux, regurgitation, and diet intolerance. It is classified into 2 subtypes: helical and nonhelical. Although stent placement and pneumatic dilation may help with nonhelical stenosis, they often do not produce sufficient results for helical stenosis. Recently, a modified gastric peroral endoscopic myotomy (G-POEM) technique (tunneling stricturotomy) has been introduced as a salvage treatment for GSS.

**Methods:**

In this case series, we present 3 cases of patients with helical sleeve stenosis treated with modified G-POEM as the initial approach. All patients in this series underwent under–saline solution tunneling, which helped navigate a swirling, challenging submucosal space. The article offers techniques and tips for successfully completing the procedure.

**Results:**

The procedure was successfully performed in all patients, who experienced significant clinical improvement and were able to tolerate a low-residue diet. No adverse events were reported.

**Conclusions:**

Although our case series demonstrates that tunneling stricturotomy was safe and effective as a primary treatment for gastric sleeve helical stenosis, additional data are needed to evaluate its safety and long-term effectiveness compared with other endoscopic treatments.

## Background and aims

Laparoscopic sleeve gastrectomy (LSG) is the most commonly performed bariatric surgery worldwide.^1^ Gastric sleeve stenosis (GSS) occurs in 0.7% to 4% of cases^2^ and is classified into sleeve helical stenosis (SHS) and nonhelical stenosis (SNHS).^3^ There is no consensus on the best treatment. Options range from medications and dietary adjustments in mild cases to endoscopic stent placement,^4^^,^^5^ pneumatic dilation,^6^^,^^7^ revision sleeve gastrectomy, surgical seromyotomy, gastropexy,^8^ and conversion to Roux-en-Y gastric bypass for severe cases.^9^^,^^10^ Endoscopy offers a less-invasive option. Although stretching techniques, such as stent placement and pneumatic dilation, may relieve symptoms in SNHS, they do not provide sufficient remodeling to effectively treat SHS.^3^ Recently, a modified gastric peroral endoscopic myotomy (G-POEM) technique (tunneling stricturotomy) has been introduced as a salvage method for GSS.^3^^,^^11^^,^^12^ This technique is more challenging than other peroral endoscopic myotomy procedures because of the rotation of the gastric wall and the limited fibrotic submucosal (SM) space at the angulation.

The saline immersion therapeutic endoscopy (SITE) technique^13^^,^^14^ can mitigate this challenge by continuously expanding the SM space, improving visualization, and stabilizing the gastroscope.

## Patients and methods

We present 3 cases of patients with SHS who underwent modified G-POEM as the initial treatment, using the SITE technique (Video 1, available online at www.videogie.org). Discussions were held with patients and the surgical team about the risks and benefits of various endoscopic and surgical options, including pneumatic dilation, stent placement, modified G-POEM, and conversion to a Roux-en-Y gastric bypass. A multidisciplinary decision was made to proceed with the modified G-POEM. All procedures were performed with the patient under general anesthesia after a prophylactic dose of antibiotics was administered, with carbon dioxide (CO_2_) used for insufflation as needed.

A viscous solution was injected into the SM, followed by a horizontal mucosal incision with a HYBRIDknife flex I-Type (Erbe, Tübingen, Germany) 4 to 5 cm proximal to the angulation. The tunnel was entered, and tunneling was performed using the SITE technique. Blood vessels were coagulated using the same knife in the preciseSECT mode for small vessels and with Coagraspers (Olympus, Tokyo, Japan) in the SOFT COAG mode for larger vessels. At the angulation, the SM space narrowed and became swirly. The SITE technique helped navigate a narrow, swirling SM space. The tunnel measured 8 to 9 cm, including a 2-cm extension beyond the angulation. A full-thickness myotomy of 6 to 7 cm was performed in a distal-to-proximal direction, involving the entire helical twist and extending an additional 2 cm proximally and distally from the angulation. Finally, the mucosotomy was closed using an OverStitch device (Boston Scientific, Marlborough, Mass, USA).

### Patient 1

**Figure 1:**
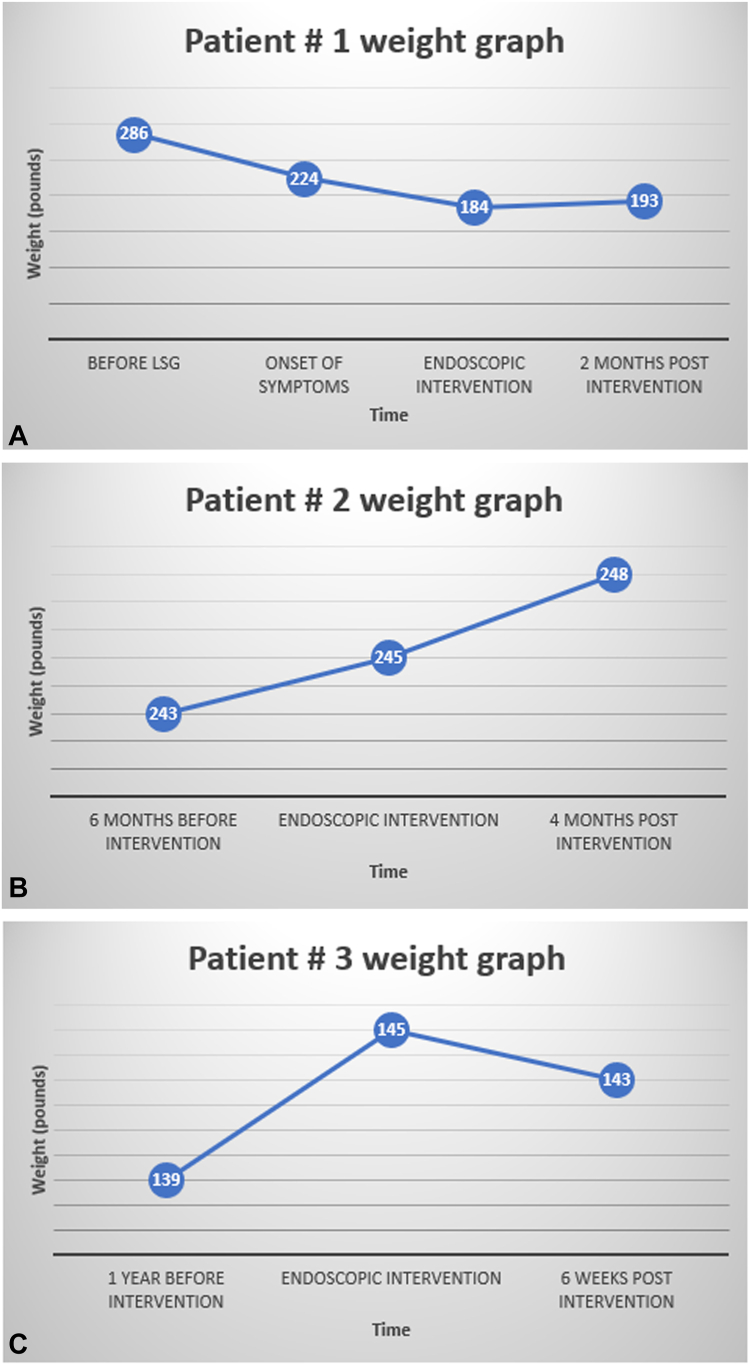
Weight graph for **(A)** patient 1, **(B)** patient 2, and **(C)** patient 3.

**Figure 2:**
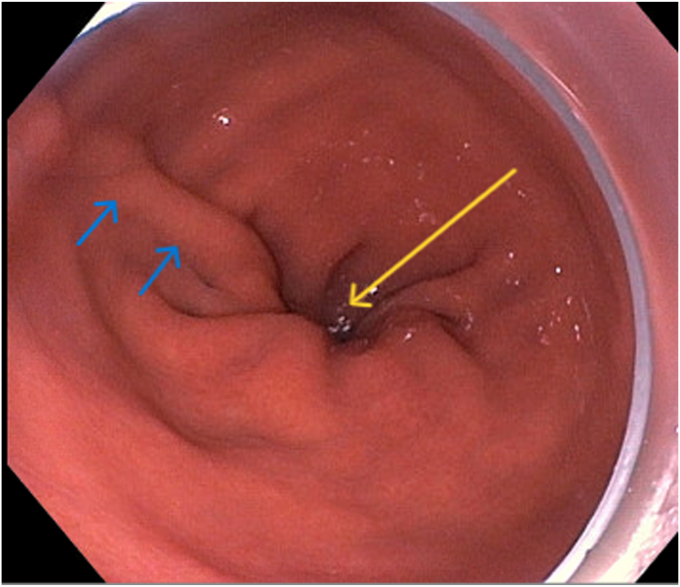
Patient 1. Endoscopic view of the helical stenosis (*yellow arrow*) and the staple line (*blue arrows*).

A 53-year-old man underwent LSG 10 months previously. Two months after surgery, he experienced severe regurgitation and reflux. He could only tolerate liquids and lost an additional 40 pounds (Table 1, Fig. 1A). An upper GI (UGI) series showed significant reflux. High-resolution manometry revealed an integrated relaxation pressure of 15.1 mm Hg. Peristalsis was weak (18% failing, 55% weak weak, and 73% ffective). The distal contractile integral was 470 mm mercury per second per cm. He was referred for suspected achalasia. An upper endoscopy revealed esophagitis and a helical angulation at the incisura (Fig. 2), navigated with sharp torque. After a multidisciplinary discussion with the patient and the surgical team, a decision was made to proceed with modified G-POEM.

**Table 1:** Eckardt scores

Patient no.	Dysphagia	Regurgitation	Retrosternal chest pain	Weight loss	Total Eckardt score
1	0	3	0	3	6
2	0	3	0	0	3
3	0	3	0	0	3

**Figure 3:**
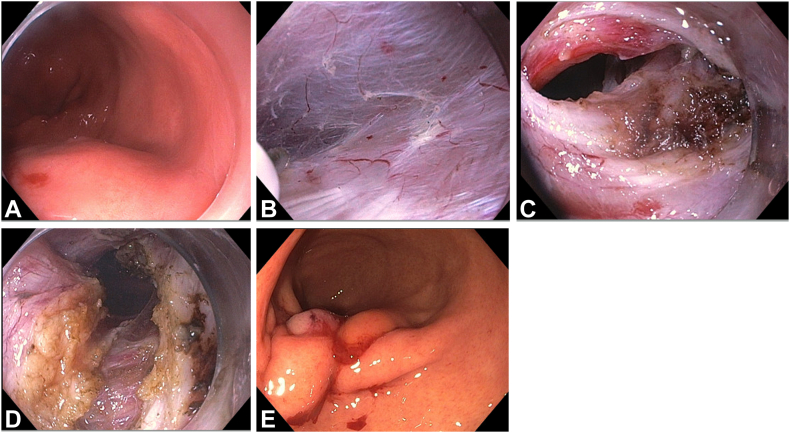
Patient 1. **A**, Submucosal injection proximal to the stenosis. **B**, Submucosal dissection under saline solution. **C**, Endoscopic view of the gastric angulation inside the tunnel after completing the submucosal tunneling. **D**, A 7-cm full-thickness myotomy was completed. **E**, The tunnel entry was closed with OverStitch suturing (Boston Scientific, Marlborough, Mass, USA).

The procedure was performed with the patient under general anesthesia and after giving a dose of antibiotics. For insufflation, CO_2_ was used as needed. Other procedural details were outlined earlier (Fig. 3, Table 2). There were no adverse events. The following day, an abdominal CT with oral contrast confirmed good closure, his diet was advanced, and he was discharged. On follow-up 2 months later, his symptoms were resolved, and he was tolerating a regular diet.

**Table 2:** Procedural details

Patient no.	Mucosal incision, cm from stenosis	Myotomy length, cm	Percutaneous decompression	Tunnel closure
1	5	7	No	Suturing
2	5	6	No	Suturing + clips
3	4	6	No	Suturing

### Patient 2

**Figure 4:**
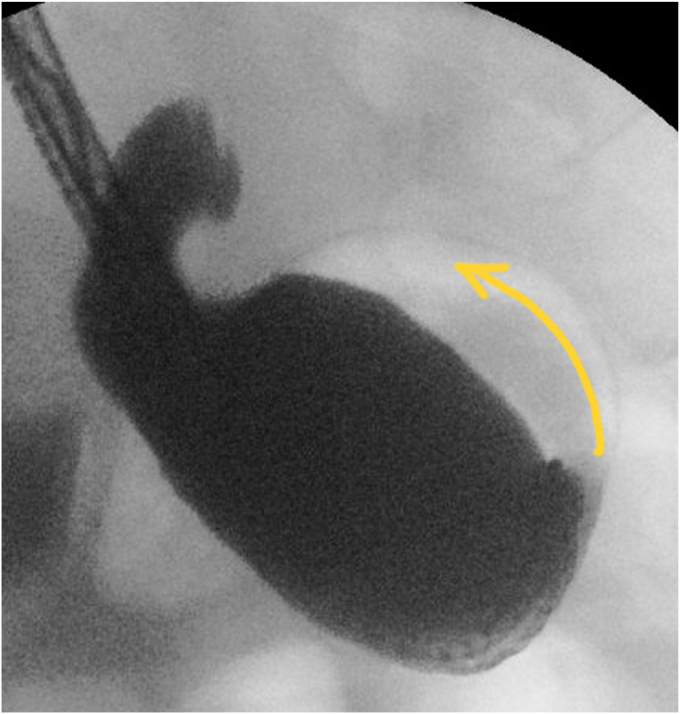
Patient 2. Contrast injection demonstrating retained contrast proximal to a helical angulation. The *yellow arrow* highlights the route leading to the antrum.

A 57-year-old man underwent LSG 10 years previously. Six months later, he had multiple emergency room visits because of nausea and vomiting. Two years after surgery, he visited his surgeon because of severe reflux. An upper endoscopy revealed esophagitis and a 4-cm hiatal hernia. He underwent laparoscopic hiatal hernia repair. Two years later, he experienced persistent vomiting and severe reflux despite taking proton pump inhibitors (PPIs). Since then, he had been on a liquid diet but managed to maintain his weight with high-calorie drinks (Table 1, Fig. 1B). He saw multiple gastroenterologists, had numerous tests, and was treated with acid suppressants, antiemetics, and diet modifications without improvement. He presented to our institution with unchanged symptoms after 6 years on a liquid diet. An upper endoscopy revealed grade D esophagitis, a small hiatal hernia, and severe twisting at the incisura, navigated with sharp torque. Intraprocedural contrast injection revealed a helical angulation with delayed contrast passage to the antrum (Fig. 4). He could not tolerate a gastric-emptying study.

**Figure 5:**
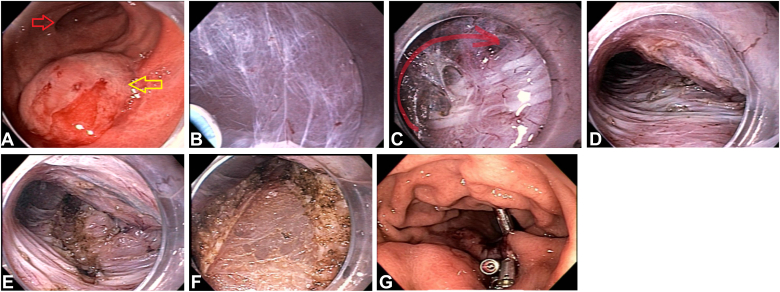
Patient 2. **A**, Submucosal injection with a viscous solution (*yellow arrow*) performed 5 cm proximal to the angulation (*red arrow*). **B**, Submucosal tunneling under saline solution. **C**, A twisted, narrowed submucosal space (*red arrow*) at the gastric angulation. **D**, Endoscopic view of the gastric angulation inside the tunnel after completing the submucosal tunneling. **E**, Starting the myotomy 2 cm distal to the gastric angulation. **F**, A 6-cm full-thickness myotomy was completed. **G**, The tunnel entry was closed with OverStitch suturing and clips (Boston Scientific, Marlborough, Mass, USA).

After a multidisciplinary discussion, a decision was made to proceed with a modified G-POEM. The procedure was performed with the patient under general anesthesia and after giving a dose of antibiotics. For insufflation, CO_2_ was used as needed. Other procedural details were outlined earlier (Fig. 5, Table 2). There were no adverse events. A UGI series 40 hours later confirmed good closure. Diet was advanced, and he was discharged within 72 hours after the procedure. On a 4-month follow-up, his symptoms had resolved, and he was tolerating a low-residue diet. Endoscopy revealed healing of esophagitis and improved angulation.

### Patient 3

A 48-year-old woman underwent LSG 15 years ago. Four years later, she underwent laparoscopic sleeve revision because of weight regain. Over the past 11 years, she experienced regurgitation, intermittent vomiting, and refractory reflux, forcing her to sleep upright. Her weight remained stable (Table 1, Fig. 1C). She saw multiple gastroenterologists and was treated with PPIs without improvement. High-resolution manometry was ordered by the surgical team in preparation for antireflux surgery, but it was not tolerated.

She was referred to us for an endoluminal functional lumen imaging probe. A UGI series showed a small hiatal hernia with severe reflux.

**Figure 6:**
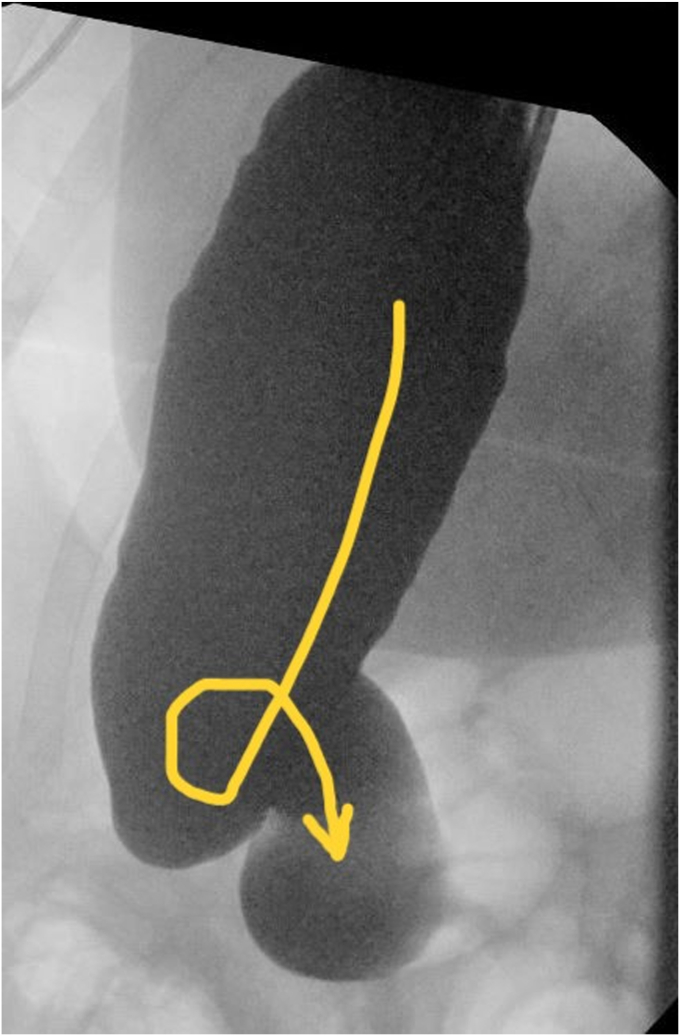
Patient 3. Contrast injection demonstrating helical angulation. The *yellow arrow* highlights the route leading to the antrum.

Upper endoscopy revealed esophagitis, a relaxed lower esophageal sphincter, and a twist in the gastric body, which was navigated with sharp torque. Intraprocedural contrast injection demonstrated pooling of contrast in the proximal stomach, followed by slow passage through a helical rotation to the antrum (Fig. 6).

**Figure 7:**
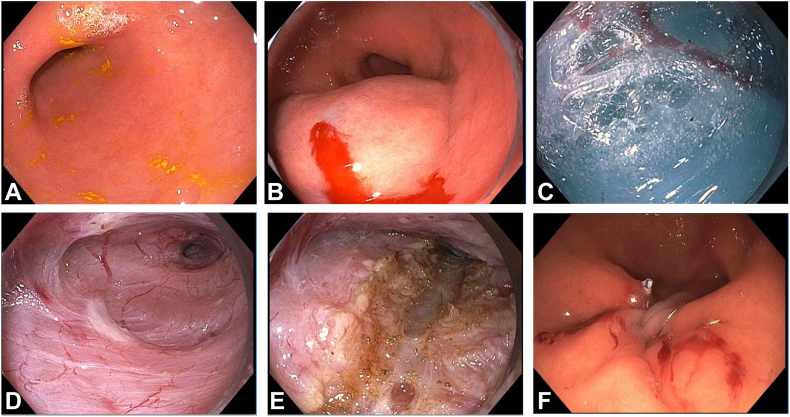
Patient 3. **A**, Endoscopic view of the helical stenosis. **B**, Submucosal injection proximal to the stenosis. **C**, A submucosal tunnel is entered. **D**, Endoscopic view of the gastric angulation inside the tunnel after completing the submucosal tunneling. **E**, A 6-cm myotomy was completed. **F**, The tunnel entry was closed with OverStitch suturing (Boston Scientific, Marlborough, Mass, USA).

After a multidisciplinary discussion, a decision was made to proceed with a modified G-POEM. The procedure was performed with the patient under general anesthesia and after giving a dose of antibiotics. For insufflation, CO_2_ was used as needed. Other procedural details were outlined earlier (Fig. 7, Table 2). During myotomy, she developed abdominal distention requiring percutaneous abdominal decompression using a 20-gauge needle.

Otherwise, there were no adverse events. A UGI series 40 hours later confirmed good closure. Diet was advanced, and she was discharged within 48 hours after the procedure. On a 6-week follow-up, her symptoms improved, and she was tolerating a low-residue diet.

## Results

All procedures were technically successful with no adverse events. A 24- to 48-hour postprocedural contrast study confirmed successful closure. All patients were discharged within 48 hours, except for patient 2, who was discharged on the third postoperative day for social reasons (Table 3).

**Table 3:** Postoperative imaging modality, imaging timing, and discharge

Patient no.	Postoperative imaging	Timing of imaging	Timing of discharge
1	CT with oral contrast	POD 1	POD 1
2	UGI series	POD 2	POD 3 (social reasons)
3	UGI series	POD 2	POD 2

*POD*, Postoperative day; *UGI*, upper GI.

At follow-up, which ranged from 6 weeks to 4 months after the procedure (Table 4), all patients showed significant improvement and could tolerate a low-residue diet.

**Table 4:** Postprocedure follow-up

Patient no.	Latest follow-up	Follow-up EGD
1	2-mo postop	NA
2	4-mo postop	4-mo postop
3	6-wk postop	NA

*NA*, Not applicable; *postop*, postoperative.

The only patient in this case series who experienced significant weight loss due to his symptoms gained weight after the procedure (Fig. 1).

## Discussion

Helical sleeve stenosis is characterized by axial rotation of the gastric tube caused by staple line spiraling in the midgastric body at the incisura,^15^ resulting in a functional blockage and increased intragastric pressures. This can lead to early disruption of the staple line, resulting in leaks or fistulas.^7^^,^^16^^,^^17^ Other symptoms include regurgitation, refractory GERD, and inability to tolerate food.^18^

Multiple mechanisms are involved, including the increased susceptibility of the remnant stomach to twisting after separation from the omentum, a spiraling pattern of stapling, and the formation of adhesions.^19^

This condition is often underdiagnosed. Patients frequently present with years of refractory GERD and nonhealing esophagitis despite treatment with PPIs and the lack of a significant hiatal hernia. This may contribute to the high prevalence of Barrett's esophagus (11%) in this population.^20^ Regurgitation can mimic achalasia. Patients may seek multiple opinions, undergo numerous tests, and some might have antireflux surgery without experiencing relief.

During endoscopy, a clockwise rotation of the staple line is observed, resulting in varying degrees of luminal narrowing. The gastroscope passes through the twisted section with a torquing maneuver without significant difficulty. This may explain why this functional obstruction is often overlooked.

CT shows a kink in the midgastric body and occasionally demonstrates the rotation of the staple line.^18^ A UGI series reveals retention of contrast above the twisted incisura, with reflux of contrast into the esophagus.^18^ A modified G-POEM technique was recently introduced to treat GSS, in which SM tunneling is performed, followed by myotomy at the twisted segment, resembling surgical seromyotomy (an effective but underused method because of its technical complexity and high postoperative leak rate^21^).

Although stent placement and dilation may help with nonhelical stenosis, these techniques do not cause sufficient muscle tearing. The modified G-POEM allows a full-thickness myotomy, promoting remodeling in cases of helical stenosis.

Zhang et al^12^ reported a case series of 13 patients treated with this technique, 85% of whom had helical stenosis. Notably, 77% did not respond to previous pneumatic dilations. The clinical success rate with the modified G-POEM was 77%, with only 23% requiring conversion to Roux-en-Y gastric bypass. There were no major adverse events. This may be because G-POEM is a controlled myotomy performed within an SM tunnel, providing an additional layer of protection. Pneumatic dilation, in contrast, carries a 1.5% risk of perforation, which is frequently sudden, large, and transmural, often requiring emergency conversion to gastric bypass surgery.^22^

Our SM tunnel was made on the posterior wall of the greater curvature, facilitating easier tunnel entry and avoiding the surgical staples.

The challenge of creating a twisting tunnel within a wispy, fibrotic SM layer was tackled in these ways:

1. Using SITE to continuously expand the SM space and protect the mucosa. In addition, the magnification effect of water made it easier to identify blood vessels.
2. Maintaining the dissection plane just above the muscularis propria enabled the tunnel to follow the spiraling rotation of the sleeve.
3. Periodically exiting the tunnel to assess its direction.
4. Keeping the tunnel wide facilitated the assessment of its overall path and prevented tunneling through the muscle.
5. Meticulously preventing the coagulation of blood vessels to help maintain visibility inside the tunnel.

CO_2_ insufflation was used during myotomy for the following reasons:

1. To prevent liquid spillage into the peritoneum during full-thickness myotomy.
2. We prefer using coagulation current with CO_2_ insufflation over the SITE with endoCUT (Erbe, Marietta, Ga, USA) for dividing the muscularis propria to decrease the risk of bleeding at deeper levels.

The full-thickness myotomy targeted the twisted area, starting 2 cm distal to the tunnel entry and extending 2 cm beyond the end of the twist.

The myotomy was initiated distally and progressed proximally in a stepwise manner for the following reason: the gastroscope shaft exerts pressure on the gastric wall as it passes through the helical stenosis. Starting a full-thickness myotomy proximally may hinder scope advancement and complicate distal completion.

## Conclusions

Post-LSG helical stenosis is an under-recognized condition that can cause prolonged symptoms, lead to misdiagnosis, and sometimes result in unnecessary antireflux surgery. Although limited data are available on modified G-POEM for treating GSS, our case series is unique in that it specifically focuses on patients with helical stenosis, using modified G-POEM as the primary endoscopic treatment. It also provides technical tips for challenging tunneling, particularly the use of SITE, which proved essential for navigating an angulated and complex SM space within the helical stenosis.

Nonetheless, more data are necessary to compare the safety and long-term effectiveness of this approach with other endoscopic techniques.

## Disclosure

All authors disclosed no financial relationships.

## Supplementary data

Video 1 Modified gastric peroral endoscopic myotomy to treat sleeve gastrectomy helical stenosis.
